# Supplementary material for: Universal Scaling in the Branching of the Tree of Life
Source: PLoS One. 2008 Jul 23;3(7):e2757. doi: 10.1371/journal.pone.0002757 (PMC2447175; doi:10.1371/journal.pone.0002757)
Supplement: Text S1 — Scaling of branch size and cumulative branch size: TreeBASE vs. manually selected data sets. We provide the list of references corresponding to the selected intraspecific and interspecific phylogenetic trees; the statistics of all data sets with two specific examples; and a summary table of taxa in the data sets. (0.06 MB DOC) [file pone.0002757.s001.doc]

**SUPPORTING INFORMATION**

***Scaling of branch size and cumulative branch size: TreeBASE vs. manually selected data sets***

The interspecific data set analyzed in this paper consists of 5212 phylogenetic trees downloaded from TreeBASE (http://www.treebase.org). Given that a database similar to TreeBASE does not exist for intraspecific phylogenies, we constructed our intraspecific data set by manually compiling 67 phylogenetic trees from several published references [S1-S45]. The difference in size between the two data sets calls for some additional checking on the appropriateness of a comparison between them. As a way to close the gap between the two datasets we compiled a third set of trees consisting of phylogenies of interspecific character, like the data in TreeBASE, but manually extracted from published references [S46-85] following the same criteria as the intraspecific set analyzed in the paper, and with the same size, 67 trees. We remind (see main text) that our selection criteria insure that our tree datasets contained organisms from terrestrial, marine and fresh water environments, from all the main climatic regions, from all kingdoms (Table S1), and reconstructed with the main phylogenetic tree estimation methods.

The results of this analysis are shown in Figure S1 (we illustrate tree structures and the allometric scaling for one intraspecific and one interspecific tree in Figures S2 and S3, respectively). It displays the cumulative complementary distribution functions (CCDFs) for branch size (*F*(*A*), panel a) and cumulative branch size (*F*(*C*), panel b), and the allometric scaling relation ($C\text{\textasciitilde}A^{\eta}$, panel c) averaged and logarithmically binned over all phylogenetic trees. We see that, despite their different size, the two interspecific data sets display the same behavior. Any bias in the manual selection procedure with respect to TreeBASE, if present, is weak enough to have no impact on the topological scaling behavior. In addition, there is perfect agreement between the scaling of the three data sets, except for the largest tree sizes for which there is poor statistics in the smaller data sets. This gives further support to the universality of the scaling found.

**Table S1. Break-down of the number of analyzed inter- and intra-species trees with respect to taxa.**

**Figure S1.** **Scaling relations from the enlarged data set described in Supporting Information.** Cumulative complementary distribution functions (CCDFs) for branch size (*F(A)*, panel A) and cumulative branch size (*F(C)*, panel B), and the allometric scaling relation ($C\text{\textasciitilde}A^{\eta}$, panel C) averaged and logarithmically binned over all phylogenetic trees. Empty squares are for the interspecific TreeBASE data set, solid circles are for the manually compiled intraspecific data set, and triangles are for the new manually compiled interspecific data set of reduced size. Solid lines are power laws fitted to the TreeBASE behavior, as in Figs. 2 and 3 of the main text.

**Figure S2. Intraspecific phylogenetic tree.** A: An example of an intraspecific phylogenetic tree: different strains of the bacteria *Vibrio vulnificus* [S19]. Most of the branchings are binary, but there are some 3rd order branchings. B: The allometric scaling plot showing the relationship of cumulative branch size (*C*) to branch size (*A*) from each node of that tree. The solid line corresponds to the fitting *C~A^1.43^* to this intraspecific dataset.

**Figure S3. Interspecific phylogenetic tree.** A: An example of an interspecific phylogenetic tree: the catfish species (order *Siluriformes*) [S80]. Most of the branchings are binary, but there are some 3rd order branchings. B: The allometric scaling plot showing the relationship of cumulative branch size (*C*) to branch size (*A*) from each node of that tree. The solid line corresponds to the fitting *C~A^1.44^* to this intraspecific dataset.

**Intraspecific and interspecific data sets**

The intraspecific and interspecific phylogenies which have been analyzed, in addition to the ones from TreeBase, have been obtained from the following references:

***Intraspecific phylogenies***

1. Albach DC, Schönswetter P, Tribsch A (2006) Comparative phylogeography of the Veronica alpina complex in Europe and North America. Mol. Ecol. 15**:** 3269-3286.
2. Beszteri B, Ács E, Medlin, LK (2005) Ribosomal DNA Sequence Variation among Sympatric Strains of the Cyclotella meneghiniana Complex (Bacillariophyceae) Reveals Cryptic Diversity. Protist 156: 317-333.
3. Choi Y-J, Hong S-B, Shin H-D (2006) Genetic diversity within the Albugo candida complex (Peronosporales, Oomycota) inferred from phylogenetic analysis of ITS rDNA and COX2 mtDNA sequences. Mol. Phylogenet. Evol. 40: 400–409.
4. Cupolillo E, Brahim LR, Toaldo CB, de Oliveira-Neto MP, de Brito MEF, Falqueto A, Naiff MdeF, Grimaldi GJr (2003) Genetic Polymorphism and Molecular Epidemiology of Leishmania (Viannia) braziliensis from Different Hosts and Geographic Areas in Brazil. J. Clin. Microbiol. 41: 3126–3132.
5. de Casas RR, Besnard G, Schönswetter P, Balaguer L, Vargas P (2006) Extensive gene flow blurs phylogeographic but not phylogenetic signal in Olea europaea L. Theor. Appl. Genet. 113: 575–583.
6. Devitt TJ (2006) Phylogeography of the Western Lyresnake (Trimorphodon biscutatus): testing aridland biogeographical hypotheses across the Nearctic–Neotropical transition. Mol. Ecol. 15: 4387–4407.
7. Driscoll DA, Hardy CM (2005) Dispersal and phylogeography of the agamid lizard Amphibolurus nobbi in fragmented and continuous habitat. Mol. Ecol. 14: 1613–1629.
8. Ehling-Schulz M, Svensson B, Guinebretiere M-H, Lindbäck T, Andersson M, Schulz A, Fricker M, Christiansson A, Granum PE, Märtlbauer E, Nguyen-The C, Salkinoja-Salonen M, Scherer S (2005) Emetic toxin formation of Bacillus cereus is restricted to a single evolutionary lineage of closely related strains. Microbiology 151: 183–197.
9. Gottschling M, Köhler A, Stockfleth E, Nindl I (2007) Phylogenetic analysis of beta-papillomaviruses as inferred from nucleotide and amino acid sequence data. Mol. Phylogenet. Evol. 42: 213–222.
10. Hahn MW, Pöckl M, Wu QL (2005) Low Intraspecific Diversity in a Polynucleobacter Subcluster Population Numerically Dominating Bacterioplankton of a Freshwater Pond. Appl. Environ. Microbiol. 71: 4539–4547.
11. Heilveil JS, Berlocher SH (2006) Phylogeography of postglacial range expansion in Nigronia serricornis Say (Megaloptera: Corydalidae). Mol. Ecol. 15: 1627–1641.
12. Hommais F, Pereira S, Acquaviva C, Escobar-Páramo P, Denamur E (2005) Single-Nucleotide Polymorphism Phylotyping of Escherichia coli. Appl. Environ. Microbiol. 71: 4784–4792.
13. Huang S, Chiang YC, Schaal BA, Chou CH, Chiang TY (2001) Organelle DNA phylogeography of Cycas taitungensis, a relict species in Taiwan. Mol. Ecol. 10: 2669-2681.
14. Humbert JF, Duris-Latour D, Le Berre B, Giraudet H, Salençon MJ (2005) Genetic Diversity in Microcystis Populations of a French Storage Reservoir Assessed by Sequencing of the 16S-23S rRNA Intergenic Spacer. Microb. Ecol. 49: 308–314.
15. Jensen LH, Enghoff H, Frydenberg J, Parker EDJr (2002) Genetic diversity and the phylogeography of parthenogenesis: comparing bisexual and thelytokous populations of Nemasoma aricorne (Diplopoda: Nemasomatidae) in Denmark.Hereditas 136: 184–194.
16. Kawamoto Y, Shotake T, Nozawa K, Kawamoto S, Tomari K-I, Kawai S, Shirai K, Morimitsu Y, Takagi N, Akaza H, Fujii H, Hagihara K, Aizawa K, Akachi S, Oi T, Hayaishi S (2007) Postglacial population expansion of Japanese macaques (Macaca fuscata) inferred from mitochondrial DNA phylogeography. Primates 48: 27-40.
17. Ko KS, Lee HK, Park MY, Kook YH (2003) Mosaic Structure of Pathogenicity Islands in Legionella pneumophila. J. Mol. Evol. 57: 63–72.
18. Lefébure T, Douady CJ, Gouy M, Trontelj P, Briolay J, Gibert J (2006) Phylogeography of a subterranean amphipod reveals cryptic diversity and dynamic evolution in extreme environments. Mol. Ecol. 15: 1797–1806.
19. Lin M, Payne DA, Schwarz JR (2003) Intraspecific Diversity of Vibrio vulnificus in Galveston Bay Water and Oysters as Determined by Randomly Amplified Polymorphic DNA PCR. Appl. Environ. Microbiol. 69: 3170–3175.
20. Liu J-X, Gao T-X, Zhuang Z-M, Jin X-S, Yokogawa K, Zhang Y-P (2006) Late Pleistocene divergence and subsequent population expansion of two closely related fish species, Japanese anchovy (Engraulis japonicus) and Australian anchovy (Engraulis australis). Mol. Phylogenet. Evol. 40: 712–723.
21. Marmi J, López-Giráldez F, MacDonald DW, Calafell F, Zholnerovskaya E, Domingo-Roura X (2006) Mitochondrial DNA reveals a strong phylogeographic structure in the badger across Eurasia. Mol. Ecol. 15: 1007–1020.
22. Marimon R, Gené J, Cano J, Trilles L, Lazéra MS, Guarro J (2006) Molecular Phylogeny of Sporothrix schenckii. J. Clin. Microbiol. 44: 3251–3256.
23. Martínez-Solano I, Teixeira J, Buckley D, García-París M (2006) Mitochondrial DNA phylogeography of Lissotriton boscai (Caudata, Salamandridae): evidence for old, multiple refugia in an Iberian endemic. Mol. Ecol. 15: 3375–3388.
24. Michitaka K, Tanaka Y, Horiike N, Duong TN, Chen Y, Matsuura K, Hiasa Y, Mizokami M, Onji M (2006) Tracing the History of Hepatitis B Virus Genotype D in Western Japan. J. Med. Virol. 78: 44–52.
25. Miller CR, Waits LP, Joyce P (2006) Phylogeography and mitochondrial diversity of extirpated brown bear (Ursus arctos) populations in the contiguous United States and Mexico. Mol. Ecol. 15: 4477–4485.
26. Monis PT, Andrews RH, Mayrhofer G, Ey PL (2003) Genetic diversity within the morphological species Giardia intestinalis and its relationship to host origin. Infect. Genet. Evol. 3: 29–38.
27. Ozeki M, Isagi Y, Tsubota H, Jacklyn P, Bowman DMJS (2007) Phylogeography of an Australian termite, Amitermes laurensis (Isoptera, Termitidae), with special reference to the variety of mound shapes. Mol. Phylogenet. Evol. 42: 236–247.
28. Perneel M, Tambong JT, Adiobo A, Floren C, Saborío F, Lévesque A, Höfte M (2006) Intraspecific variability of Pythium myriotylum isolated from cocoyam and other host crops. Mycol. Res. 110: 583 – 593.
29. Perk S, Banet-Noach C, Shihmanter E, Pokamunski S, Pirak M, Lipkind M, Panshina A (2006) Genetic characterization of the H9N2 influenza viruses circulated in the poultry population in Israel. Comp. Immunol. Microbiol. Infect. Dis. 29: 207–223.
30. Roberts TE (2006) History, ocean channels, and distance determine phylogeographic patterns in three widespread Philippine fruit bats (Pteropodidae). Mol. Ecol. 15: 2183–2199.
31. Rowe KC, Heske EJ, Paige KN (2006) Comparative phylogeography of eastern chipmunks and white-footed mice in relation to the individualistic nature of species. Mol. Ecol. 15: 4003–4020.
32. Ruzzante DE, Walde SJ, Cussac VE, Dalebout ML, Seibert J, Ortubay S, Habit E (2006) Phylogeography of the Percichthyidae (Pisces) in Patagonia: roles of orogeny, glaciation, and volcanism. Mol. Ecol. 15: 2949–2968.
33. Scott JB, Chakraborty S (2006) Multilocus sequence analysis of Fusarium pseudograminearum reveals a single phylogenetic species. Mycol. Res. 110: 1413–1425.
34. Sogstad MKR, Høiby EA, Caugant DA (2006) Molecular Characterization of Non-Penicillin-Susceptible Streptococcus pneumoniae in Norway. J. Clin. Microbiol. 44: 3225–3230.
35. Thangadurai R, Hoti SL, Kumar NP, Das PK (2006) Phylogeography of human lymphatic filarial parasite, Wuchereria bancrofti in India. Acta Trop. 98: 297–304.
36. Ursenbacher S, Carlsson M, Helfer V, Tegelström H, Fumagalli L (2006) Phylogeography and Pleistocene refugia of the adder (Vipera berus) as inferred from mitochondrial DNA sequence data. Mol. Ecol. 15: 3425–3437.
37. Vancanneyt M, Huys G, Lefebvre K, Vankerckhoven V, Goossens H, Swings J (2006) Intraspecific Genotypic Characterization of Lactobacillus rhamnosus Strains Intended for Probiotic Use and Isolates of Human Origin. Appl. Environ. Microbiol. 72: 5376–5383.
38. van Ee BW, Jelinski N, Berry PE, Hipp AL (2006) Phylogeny and biogeography of Croton alabamensis (Euphorbiaceae), a rare shrub from Texas and Alabama, using DNA sequence and AFLP data. Mol. Ecol. 15: 2735–2751.
39. Verovnik R, Sket B, Trontelj P (2004) Phylogeography of subterranean and surface populations of water lice Asellus aquaticus (Crustacea: Isopoda). Mol. Ecol. 13: 1519–1532.
40. Ward TJ, Gorski L, Borucki MK, Mandrell RE, Hutchins J, Pupedis K (2004) Intraspecific Phylogeny and Lineage Group Identification Based on the prfA Virulence Gene Cluster of Listeria monocytogenes. J. Bacteriol. 186: 4994–5002.
41. Whipps CM, Kent ML (2006) Phylogeography of the Cosmopolitan Marine Parasite Kudoa thyrsites (Myxozoa: Myxosporea). J. Eukaryot. Microbiol. 53: 364–373.
42. Zhang C, Mammen MPJr, Chinnawirotpisan P, Klungthong C, Rodpradit P, Nisalak A, Vaughn DW, Nimmannitya S, Kalayanarooj S, Holmes EC (2006) Structure and age of genetic diversity of dengue virus type 2 in Thailand. J. Gen. Virol. 87: 873–883.
43. Zhang W-J, Yang J, Yu Y-H, Shu S-W, Shen Y-F (2006) Population Genetic Structure of Carchesium polypinum (Ciliophora: Peritrichia) in Four Chinese Lakes Inferred from ISSR Fingerprinting: High Diversity but Low Differentiation. J. Eukaryot. Microbiol. 53: 358–363.
44. Zink RM, Drovetski SV, Rohwer S (2006) Selective neutrality of mitochondrial ND2 sequences, phylogeography and species limits in Sitta europaea. Mol. Phylogenet. Evol. 40, 679–686.
45. Zorrilla I, Moriñigo MA, Castro D, Balebona MC, Borrego JJ (2003) Intraspecific characterization of Vibrio alginolyticus isolates recovered from cultured fish in Spain. J. Appl. Microbiol. 95: 1106–1116.

***Interspecific phylogenies***

1. Andreasen K, Bremer B (2000) Combined phylogenetic analysis in the rubiaceae-ixoroideae: morphology, nuclear and chloroplast DNA data. Am. J. Bot. 87: 1731–1748.
2. Benz B W, Robbins MB, Peterson A T (2006) Evolutionary history of woodpeckers and allies (Aves: Picidae): Placing key taxa on the phylogenetic tree. Mol. Phylogenet. Evol. 40: 389–399.
3. Brindefalk B, Viklund J, Larsson D, Thollesson M, Andersson SG (2007) Origin and Evolution of the Mitochondrial Aminoacyl-tRNA Synthetases. Mol. Biol. Evol. 24: 743-756.
4. Dighe AS, Jangid K, González JM, Pidiyar VJ, Patole, MS, Ranade DR, Shouche YS (2004**)** Comparison of 16S rRNA gene sequences of genus Methanobrevibacter. BMC Microbio. 4: 20.
5. Dohrmann M, Voigt O, Erpenbeck D, Wörheide G (2006) Non-monophyly of most supraspecific taxa of calcareous sponges (Porifera, Calcarea) revealed by increased taxon sampling and partitioned Bayesian analysis of ribosomal DNA. Mol. Phylogenet. Evol. 40: 830–843.Duda TFJr, Kohn AJ (2005) Species-level phylogeography and evolutionary history of the hyperdiverse marine gastropod genus Conus. Mol. Phylogenet. Evol. 34: 257–272.
6. Ellison NW, Liston A, Steiner JJ, Williams WM, Taylor NL (2006) Molecular phylogenetics of the clover genus (Trifolium—Leguminosae). Mol. Phylogenet. Evol. 39: 688–705.
7. Endress PK, Doyle JA (2007) Floral phyllotaxis in basal angiosperms: development and evolution. Curr. Opin. Plant Biol. 10: 52-57.
8. Fitzpatrick DA, Logue ME, Stajich JE, Butler G (2006) A fungal phylogeny based on 42 complete genomes derived from supertree and combined gene analysis. BMC Evol. Biol. 6: 99.
9. Fuchs J, Cruaud C, Couloux A, Pasquet E (2007) Complex biogeographic history of the cuckoo-shrikes and allies (Passeriformes: Campephagidae) revealed by mitochondrial and nuclear sequence data. Mol. Phylogenet. Evol. 44: 138–153.
10. Fulton TL, Strobeck C (2006) Molecular phylogeny of the Arctoidea (Carnivora): Effect of missing data on supertree and supermatrix analyses of multiple gene data sets. Mol. Phylogenet. Evol. 41: 165–181.
11. Gamage DT, de Silva MP, Inomata N, Yamazaki T, Szmidt AE (2006) Comprehensive molecular phylogeny of the sub-family dipterocarpoideae (dipterocarpaceae) based on chloroplast DNA sequences. Genes Genet. Syst. 81: 1-12.
12. García D, Stchigel AM, Cano J, Calduch M, Hawksworth DL, Guarro J (2006) Molecular phylogeny of Coniochaetales. Mycol. Res. 110: 1271–1289.
13. Garcia J-L, Patel BK, Ollivier B (2000) Taxonomic, Phylogenetic, and Ecological Diversity of Methanogenic Archaea. Anaerobe 6: 205-226.
14. Gast RJ (2006) Molecular Phylogeny of a Potentially Parasitic Dinoflagellate Isolated from the Solitary Radiolarian, Thalassicolla nucleata. J. Eukaryot. Microbiol. 53: 43–45.
15. Gaubert P, Cordeiro-Estrela P (2006) Phylogenetic systematics and tempo of evolution of the Viverrinae (Mammalia, Carnivora, Viverridae) within feliformians: Implications for faunal exchanges between Asia and Africa. Mol. Phylogenet. Evol. 41: 266–278.
16. Habaye MS, Ekengren SK, Hultmark D (2006) Nora virus, a persistent virus in Drosophila, defines a new picorna-like virus family. J. Gen. Virol. 87: 3045–3051.
17. Hahn WJ (2002) A Molecular Phylogenetic Study of the Palmae (Arecaceae) Based on atpB, rbcL, and 18S nrDNA Sequences. Syst. Biol. 51: 92–112.
18. Huang L-N, Zhu S, Zhou H, Qu L-H (2005) Molecular phylogenetic diversity of bacteria associated with the leachate of a closed municipal solid waste landfill. FEMS Microbiol. Lett. 242: 297–303.
19. Hyvönen J, Koskinen S, Merrill GL, Hedderson TA, Stenroos S (2004) Phylogeny of the Polytrichales (Bryophyta) based on simultaneous analysis of molecular and morphological data. Mol. Phylogenet. Evol. 31: 915–928.
20. Lavoué S, Miya M, Saitoh K, Ishiguro NB, Nishida M (2007) Phylogenetic relationships among anchovies, sardines, herrings and their relatives (Clupeiformes), inferred from whole mitogenome sequences. Mol. Phylogenet. Evol. 43: 1096–1105.
21. Le M, Raxworthy CJ, McCord WP, Mertz L (2006) A molecular phylogeny of tortoises (Testudines: Testudinidae) based on mitochondrial and nuclear genes. Mol. Phylogenet. Evol. 40: 517–531.
22. Li L, Song W, Warren A, Wang Y, Ma H, Hu X, Chen Z (2006) Phylogenetic position of the marine ciliate, Cardiostomatella vermiforme (Kahl, 1928) Corliss, 1960 inferred from the complete SSrRNA gene sequence, with establishment of a new order Loxocephalida n. ord. (Ciliophora, Oligohymenophorea). Eur. J. Protistol. 42: 107–114.
23. Mallatt J, Giribet G (2006) Further use of nearly complete 28S and 18S rRNA genes to classify Ecdysozoa: 37 more arthropods and a kinorhynch. Mol. Phylogenet. Evol. 40: 772–794.
24. Mann NI, Barker FK, Graves JA, Dingess-Mann KA, Slater PJ (2006) Molecular data delineate four genera of “Thryothorus” wrens. Mol. Phylogenet. Evol. 40: 750–759.
25. Maraun M, Heethoff M, Schneider K, Scheu S, Weigmann G, Cianciolo J, Thomas RH, Norton RA (2004) Molecular phylogeny of oribatid mites (Oribatida, Acari): evidence for multiple radiations of parthenogenetic lineages. Exp. Appl. Acarol. 33: 183–201.
26. Moreira D, von der Heyden S, Bass D, López-García P, Chao E, Cavalier-Smith T (2007) Global eukaryote phylogeny: Combined small- and large-subunit ribosomal DNA trees support monophyly of Rhizaria, Retaria and Excavata. Mol. Phylogenet. Evol. 44: 255–266.
27. Moyle RG, Marks BD (2006) Phylogenetic relationships of the bulbuls (Aves: Pycnonotidae) based on mitochondrial and nuclear DNA sequence data. Mol. Phylogenet. Evol. 40: 687–695.
28. Ohlson JI, Prum RO, Ericson PG (2007) A molecular phylogeny of the cotingas (Aves: Cotingidae). Mol. Phylogenet. Evol. 42: 25–37.
29. Robalo JI, Almada VC, Levy A, Doadrio I (2007) Re-examination and phylogeny of the genus Chondrostoma based on mitochondrial and nuclear data and the definition of 5 new genera. Mol. Phylogenet. Evol. 42: 362–372.
30. Sagegami-Oba R, Oba Y, Ôhira H (2007) Phylogenetic relationships of click beetles (Coleoptera: Elateridae) inferred from 28S ribosomal DNA: Insights into the evolution of bioluminescence in Elateridae. Mol. Phylogenet. Evol. 42: 410–421.
31. Saldarriaga JF, McEwan ML, Fast NM, Taylor FJ, Keeling PJ (2003) Multiple protein phylogenies show that Oxyrrhis marina and Perkinsus marinus are early branches of the dinoflagellate lineage. Int. J. Syst. Evol. Microbiol. 53: 355–365.
32. Sjölin E, Erséus C, Källersjö M (2005) Phylogeny of Tubificidae (Annelida, Clitellata) based on mitochondrial and nuclear sequence data. Mol. Phylogenet. Evol. 35: 431–441.
33. Sørensen MV, Giribet G (2006) A modern approach to rotiferan phylogeny: Combining morphological and molecular data. Mol. Phylogenet. Evol. 40: 585–608.
34. Stchigel AM, Cano J, Miller AN, Calduch M, Guarro J (2006) Corylomyces: a new genus of Sordariales from plant debris in France. Mycol. Res. 110: 1361–1368.
35. Sullivan JP, Lundberg JG, Hardman M (2006) A phylogenetic analysis of the major groups of catfishes (Teleostei: Siluriformes) using rag1 and rag2 nuclear gene sequences. Mol. Phylogenet. Evol. 41: 636–662.
36. Wang Z, Binder M, Schoch CL, Johnston PR, Spatafora JW, Hibbett DS (2006) Evolution of helotialean fungi (Leotiomycetes, Pezizomycotina): A nuclear rDNA phylogeny. Mol. Phylogenet. Evol. 41: 295–312.
37. Wright A-D (2006) Phylogenetic relationships within the order Halobacteriales inferred from 16S rRNA gene sequences. Int. J. Syst. Evol. Microbiol. 56: 1223–1227.
38. Zanatta DT Murphy RW (2006) Evolution of active host-attraction strategies in the freshwater mussel tribe Lampsilini (Bivalvia: Unionidae). Mol. Phylogenet. Evol. 41: 195–208.
39. Zhang Z, Kudo T, Nakajima Y, Wang Y (2001) Clarification of the relationship between the members of the family Thermomonosporaceae on the basis of 16S rDNA, 16S–23S rRNA internal transcribed spacer and 23S rDNA sequences and chemotaxonomic analyses. Int. J. Syst. Evol. Microbiol. 51: 373–383.
40. Zuccon D, Cibois A, Pasquet E, Ericson PG (2006) Nuclear and mitochondrial sequence data reveal the major lineages of starlings, mynas and related taxa. Mol. Phylogenet. Evol. 41: 333–344.
