# Supplementary material for: Universal Scaling in the Branching of the Tree of Life
Source: PLoS One. 2008 Jul 23;3(7):e2757. doi: 10.1371/journal.pone.0002757 (PMC2447175; doi:10.1371/journal.pone.0002757)
Supplement: Table S1 — Break-down of the number of analyzed inter- and intra-species trees with respect to taxa. (0.03 MB DOC) [file pone.0002757.s002.doc]

|  | INTER | INTRA |
| --- | --- | --- |
| *Animalia* | 26 | 24 |
| *Archaea* | 3 | 0 |
| *Bacteria* | 9 | 18 |
| *Fungi* | 13 | 6 |
| *Plantae* | 8 | 6 |
| *Protozoa* | 6 | 4 |
| *Viruses* | 2 | 9 |

**Table S1. Break-down of the number of analyzed inter- and intra-species trees with respect to taxa.**
