# Supplementary figures and images for: Universal Scaling in the Branching of the Tree of Life
Source: PLoS One. 2008 Jul 23;3(7):e2757. doi: 10.1371/journal.pone.0002757 (PMC2447175; doi:10.1371/journal.pone.0002757)

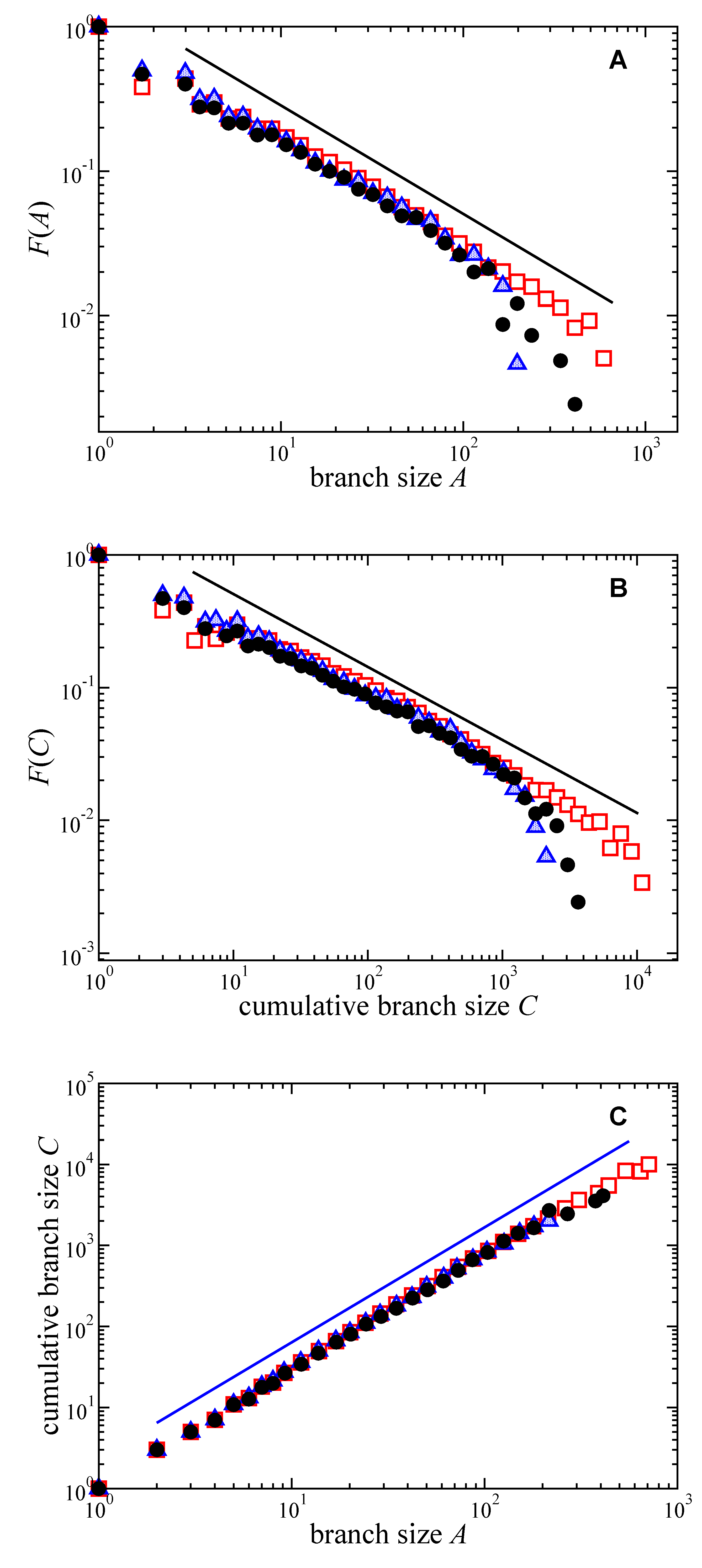

Supplement: Figure S1 — Cumulative complementary distribution functions (CCDFs) for branch size (F(A), panel A) and cumulative branch size (F(C), panel B), and the allometric scaling relation (C {similar, tilde operator } A η, panel B) averaged and logarithmically binned over all phylogenetic trees. Empty squares are for the interspecific TreeBASE data set, solid circles are for the manually compiled intraspecific data set, and triangles are for the new manually compiled interspecific data set of reduced size. Solid lines are power laws fitted to the TreeBASE behavior, as in Figs. 2 and 3 of the main text. (1.22 MB TIF) [file pone.0002757.s003.tif]

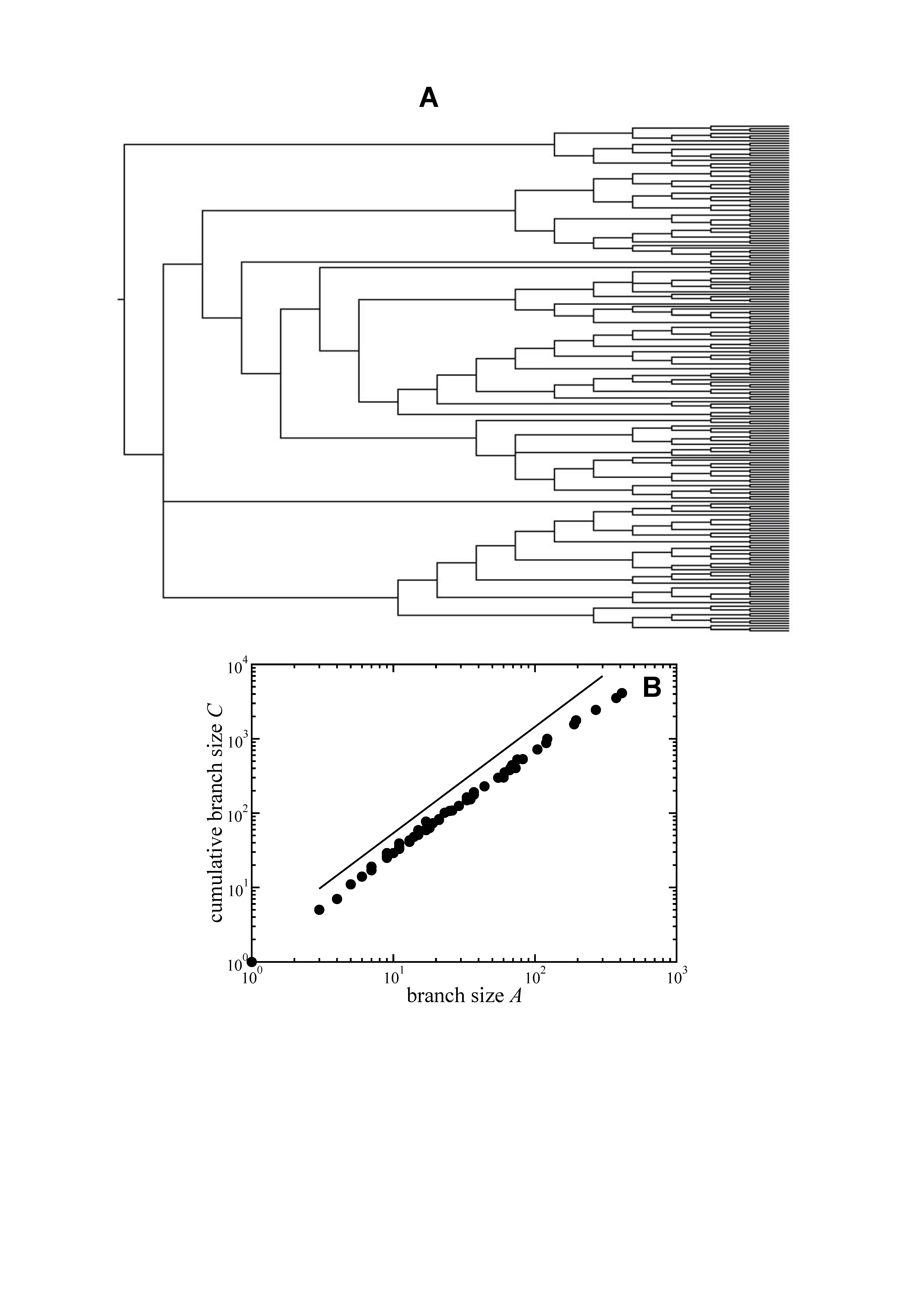

Supplement: Figure S2 — A: An example of an intraspecific phylogenetic tree: different strains of the bacteria Vibrio vulnificus [S19]. Most of the branchings are binary, but there are some 3rd order branchings. B: The allometric scaling plot showing the relationship of cumulative branch size (C) to branch size (A) from each node of that tree. The solid line corresponds to the fitting C {similar, tilde operator } A 1.43 to this intraspecific dataset. (2.66 MB TIF) [file pone.0002757.s004.tif]

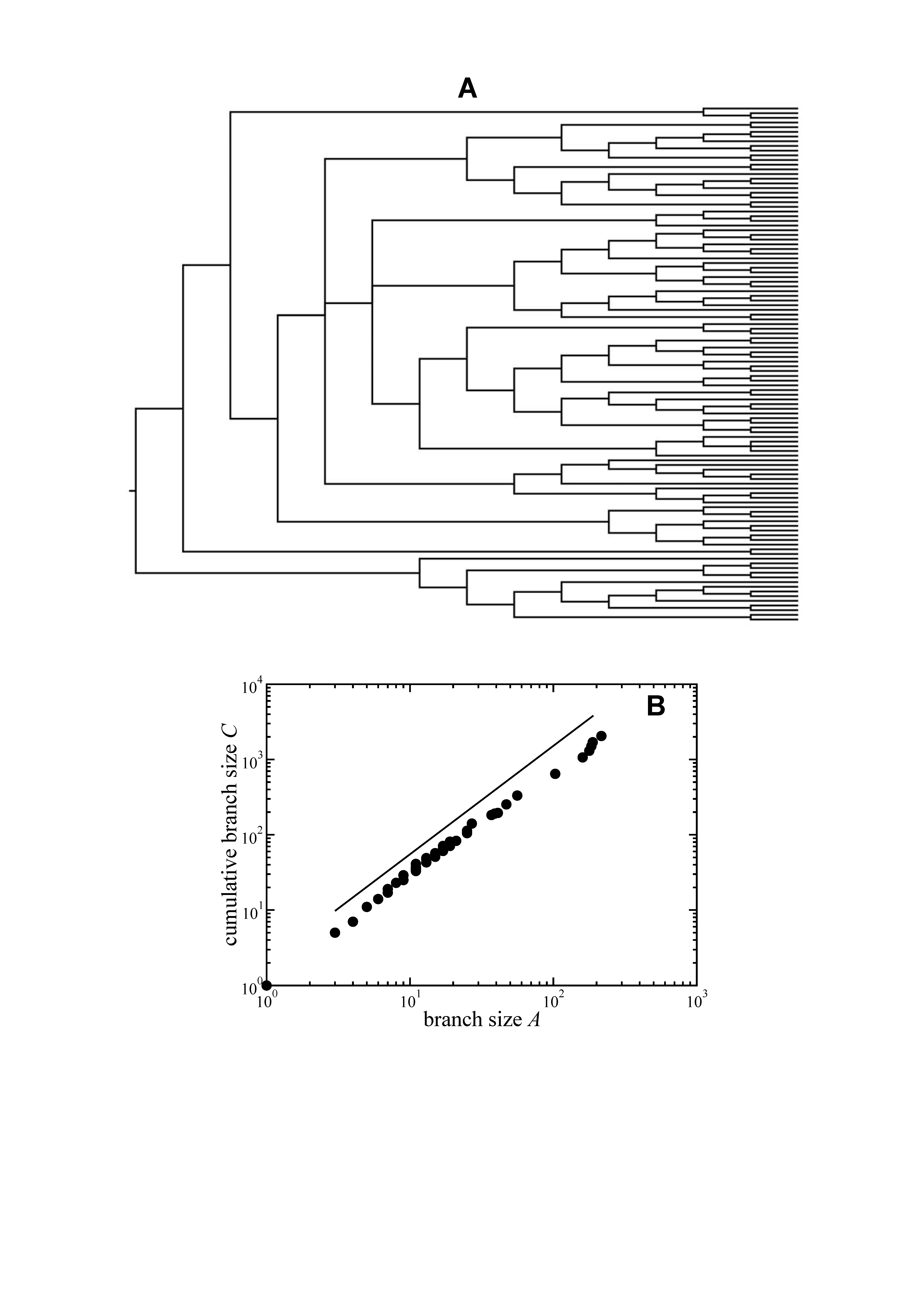

Supplement: Figure S3 — A: An example of an interspecific phylogenetic tree: the catfish species (order Siluriformes) [S80]. Most of the branchings are binary, but there are some 3rd order branchings. B: The allometric scaling plot showing the relationship of cumulative branch size (C) to branch size (A) from each node of that tree. The solid line corresponds to the fitting C {similar, tilde operator } A 1.44 to this intraspecific dataset. (2.58 MB TIF) [file pone.0002757.s005.tif]
